# Environmental Risk Assessment System for Phosphogypsum Tailing Dams

**DOI:** 10.1155/2013/680798

**Published:** 2013-12-08

**Authors:** Xin Sun, Ping Ning, Xiaolong Tang, Honghong Yi, Kai Li, Lianbi Zhou, Xianmang Xu

**Affiliations:** ^1^Faculty of Environmental Science and Engineering, Kunming University of Science and Technology, Kunming 650093, China; ^2^Department of Environmental Engineering, University of Science and Technology Beijing, Beijing 100083, China; ^3^Beijing General Research Institute of Mining and Metallurgy, Beijing 100160, China

## Abstract

This paper may be of particular interest to the readers as it provides a new environmental risk assessment system for phosphogypsum tailing dams. In this paper, we studied the phosphogypsum tailing dams which include characteristics of the pollution source, environmental risk characteristics and evaluation requirements to identify the applicable environmental risk assessment methods. Two analytical methods, that is, the analytic hierarchy process (AHP) and fuzzy logic, were used to handle the complexity of the environmental and nonquantitative data. Using our assessment method, different risk factors can be ranked according to their contributions to the environmental risk, thereby allowing the calculation of their relative priorities during decision making. Thus, environmental decision-makers can use this approach to develop alternative management strategies for proposed, ongoing, and completed PG tailing dams.

## 1. Introduction

The rapid industrialization in China has consumed vast amounts of various industrial raw materials and large quantities of industrial solid wastes remain from mining, mineral processing, and smelting processes [1]. Thousands of industrial sites are contaminated by industrial waste, which are significant threats to the environment and human health. The production and storage of industrial solid waste have resulted in land contamination and the loss of natural resources, as well as posing significant environmental risks.

Phosphogypsum (PG) is an acidic by-product of the phosphate fertilizer industry, which is produced during the production of phosphoric acid from phosphate rock. Large amounts of PG have been produced around the world and the production will increase to several hundred million metric tonnes annually [2]. Over 60 million tonnes of PG is produced per annum in China, which poses various environmental and storage problems.

Environmental risk assessments are widespread, including ecological, water, soil, and atmospheric environmental risk assessment [3–7]. At present, many experts use site-specific evaluation criteria and methods to assess different types of environmental risks in different areas, such as mine sites and urban environments. For example, ecological risk assessment guidelines have been enacted by the USA [8]. Health risk assessment has been used to evaluate brownfield sites contaminated by POPs [9]. The establishment of environmental risk assessments started relatively recently in China. At present, many areas are contaminated and serious pollution problems demand environmental risk assessments, such as PG tailing dams. Thus, we studied PG tailing dams, including the characteristics of the pollution sources and the environmental risk characteristics and identified the evaluation requirements for environmental risk assessment methods.

## 2. New Risk Assessment Approach

The current environmental risk assessment system (ERAS) is an integrated risk assessment, which considers all of the possible factors that affect the environmental risks due to pollution from PG tailing dams.

The main problem of ERAS for PG tailing dams is the integrated assessment of information from many different pollution sources, including quantitative and qualitative data. Therefore, it is necessary to develop detailed assessment methods based on the risk characteristics. Several methods have been developed for risk assessment, including life cycle assessment (LCA), safety check list (SCL), probabilistic risk assessment (PRA), and the analytic hierarchy process (AHP). AHP is one of the most widely used assessment methods. AHP is based on the premise that decision-making related to complicated problems can be handled using a hierarchical structure that transforms complexity into a simple and comprehensible problem [10, 11]. AHP has a wide range of applications, but the conventional AHP approach may not fully reflect the style of human thinking. For example, human judgment is usually represented as accurate numbers in AHP, but decision-makers usually feel more confident about giving interval judgments, rather than expressing their judgments as numeric values in actual situations [12, 13]. Therefore, AHP and fuzzy logic are used as tools to handle problems where there is high complexity, such as environmental and uncertain data. AHP can support environmental decision-makers by providing quantitative results and this ERAS approach can be applied to PG tailing dams.

**Figure 1:**
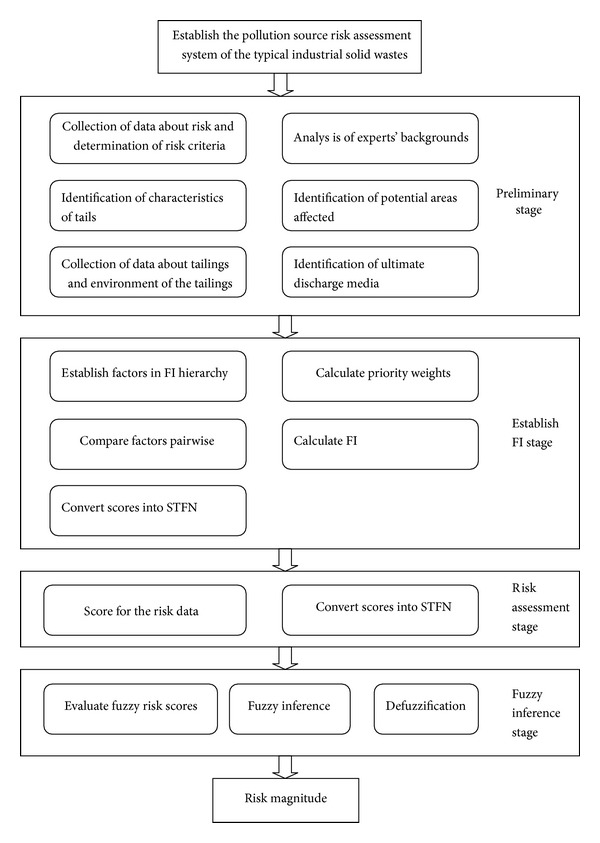
Risk assessment approach of the typical staple industrial solid waste (TSISW).

In addition, the environmental supervision of industrial solid waste is a tremendous responsibility. Thus, the establishment of ERAS for PG tailing dams is very important for solid waste management and technology systems. Due to the characteristic requirements of ERAS for PG tailing dams, fuzzy logic and AHP can be combined to provide a more comprehensive analysis. This method may be extended to the development of an ERAS for solid waste management [13]. The proposed approach is shown in Figure 1.

### 2.1. Preliminary Stage

An abundance of risk data and information are related to PG tailing dams, so the establishment of an ERAS requires a range of experts from different disciplines with essential experience in construction. During the preliminary stage, the risk assessment group collected data related to risk, determined the risk criteria, identified the characteristics of tailings, obtained data related to tailing dams and the environments of tailing dams, analyzed the backgrounds of experts, identified potentially affected areas, and identified the final discharge media, and so forth.

### 2.2. Establishment of a Factor Index (FI) Stage

#### 2.2.1. Establishment of the Factors in the FI Hierarchy

**Figure 2:**
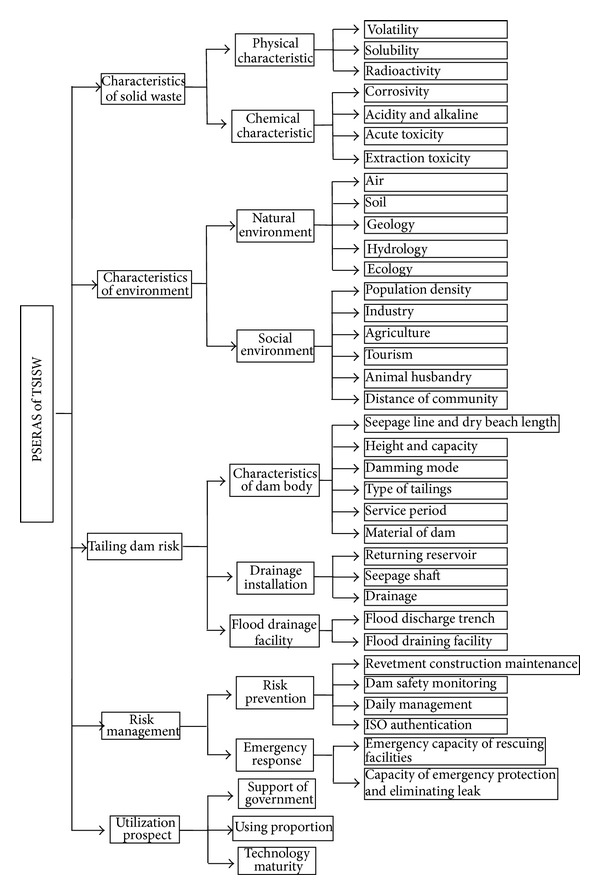


Many previous studies have shown that the AHP method can be used for multiobjective decision-making. The main sections of the overall hierarchy structure are based on the expert opinion and the qualitative analysis of the environment in the study area. The ERAS used for PG tailing dams is shown in Figure 2. In this section, we will explain the details of each level.

At the first level in the hierarchy, the ERAS of PG tailing dams is the aim of the analysis. The second level includes the solid waste characteristics, environmental characteristics, tailing dam risk, risk management, and utilization prospects.

The solid waste characteristics refer to the characteristics of PG, which are used to evaluate the risk of the solid waste itself. The environmental characteristics include the geographical position, local hydrogeology conditions, and the aspects of the surrounding environment that are sensitive to tailings. These are significant factors, which are used to evaluate the level of environmental risk, and they are also the most closely related to the production activities of humans. The tailing dam risk refers to the tailing dam's interactions with its surroundings and human activities, which is used to measure the risk of security issues related to PG tailing dams. Risk management is related to the management and maintenance of PG tailing dams. The level of risk in this system is affected by risk management, and many accidents that occur in tailing dams are due to poor supervision. The utilization prospects refer to the comprehensive utilization of PG and government support.

At the third level, the characteristics of the solid waste are subfactors based on the physical and chemical characteristic. These factors are the inherent potential risks of the pollution source. The tailing dam risk factors comprise the characteristics of the dam body, drainage installation, and flood drainage facility. The risk management factors comprise risk prevention and emergency responses. The utilization prospect factors comprise government support and using mature technology, which are vital for this system. Thus, they are placed at the third level.

Solubility, volatility, and radioactivity were selected as the indexes for the physical characteristics at the fourth level. The solubility index reflects the water solubility of the tailings and the risk of leachate outflows, which are harmful to the environment. Radioactivity reflects the risk to the environment from the radioactivity for solid waste, such as radium-226 and its subfield, thorium-232 and its subfields, and potassium-40. Corrosiveness, acidity, alkalinity, acute toxicity, and extraction toxicity were used as subfactors for the chemical characteristics. Corrosiveness reflects the possibility of impermeable membranes being corroded by pollutants. The alkalinity reflects the extent of transfer in the environment. Therefore, the chemical characteristics are very important. The acute toxicity reflects the harmful effects of pollutants on organisms in the short-term, which directly reflects the harmful extent of pollutants. The extraction toxicity is an estimated index for solid wastes, which reflects the negative extent and transfer of pollutants after solid wastes have been leached by water.

The environmental characteristics are very important because they are related to the natural environment and the social environment. The natural environment in different regions has significant effects on the migration and transformation of pollutants. For example, acidic soils will increase heavy metal pollution in most cases, while different regional environments may have different levels of risk due to pollutants with respect to the possible loss of life and property. The natural environment includes the air, soil, geology, hydrology, and ecology. The social environment includes the population density, industry, agriculture, tourism, animal husbandry, and distances between communities.

The tailing dam risk reflects the direct impact on the environment based on its degree of stability. The stability of tailings is a security issue and an environmental issue because security risks can lead to environmental pollution. The factors related to the tailing dam risk include the seepage line and dry beach length, returning reservoir, height and capacity of tailings, drainage conditions, dam construction method, flood discharge trench, and the type of tailings.

Poor risk management is the main cause of accidents. Thus, normative operations and effective management will greatly reduce the likelihood of accidents. Risk management can be divided into risk prevention and emergency responses. Risk prevention considers the prevention capacity before the accident, including revetment construction maintenance, daily dam safety monitoring, and ISO authentication. Emergency responses reflect the handling after the accident, including the emergency capacity of rescue facilities and the capacity for emergency protection and leak elimination.

The utilization prospects are the most important of factors, because they indicate the current and future comprehensive utilization situations, while they also reflect the degree of recognition and the degree of support from governments for PG tailing dams.

#### 2.2.2. Pairwise Comparisons of Factors

We conducted pairwise comparison of the factors at the same level based on their relative contributions to the ERAS. The pairwise comparisons used scores on a scale of 1–9, where 1 denoted factors with equal importance, and 3, 5, 7, and 9 denoted factors with weak, strong, very strong, and the highest importance, respectively. The experts could award scores using a fuzzy scale if necessary. The scores of the pairwise comparisons were in different formats, so we had to convert them into a common form before the calculations. Standard trapezoidal fuzzy numbers (STFN) were also used in this study [11] and the conversion equation is shown in Table 1.

#### 2.2.3. Establishment of the Pairwise Comparison Matrix

A matrix *A* was constructed according for the pairwise comparisons using the following:

(1) $$\begin{array}{c} A=\left[\begin{array}{cccc} {\tilde{x}}_{11} & {\tilde{x}}_{12} & \cdots & {\tilde{x}}_{1n}\\ {\tilde{x}}_{21} & {\tilde{x}}_{22} & \cdots & {\tilde{x}}_{2n}\\ \vdots & \vdots & \cdots & \vdots \\ {\tilde{x}}_{n1} & {\tilde{x}}_{n2} & \cdots & {\tilde{x}}_{nn} \end{array}\right], \end{array}$$

(2) $$\begin{array}{c} {\tilde{x}}_{ij}=\left(a_{ij}^{l},a_{ij}^{m},a_{ij}^{n},a_{ij}^{s}\right),\;\;{\tilde{x}}_{ji}={\tilde{x}}_{ij}^{-1}={\left(a_{ij}^{s},a_{ij}^{n},a_{ij}^{m},a_{ij}^{l}\right)}^{-1}, \end{array}$$

where ${\tilde{x}}_{ij}$ is the scale of *T*_*i*_ comparing with *T*_*j*_, while the scale is ${\tilde{x}}_{ji}$ when *T*_*j*_ comparing with *T*_*i*_.

#### 2.2.4. Consistency Checking

Before calculating the weights of the index, the consistency of the comparison matrix must be checked. To check the consistency of the comparison matrix in an intuitive manner, the fuzzy numbers are first converted into matching crisp values using the following:

(3) $$\begin{array}{c} x_{ij}=\frac{a_{ij}^{l}+2*a_{ij}^{m}+2*a_{ij}^{n}+a_{ij}^{s}}{6}. \end{array}$$

*(a) Calculate the Largest Eigenvalue of the Matrix.* The largest eigenvalue of the matrix can be calculated as follows [14]:

(4) $$\begin{array}{c} A\cdot w=\lambda_{\max}\cdot w, \end{array}$$

where *w* is the principal eigenvector of the matrix.

*(b) Consistency Check.* The consistency of the comparison matrix can be determined using the consistency ratio (CR) as follows:

(5) $$\begin{array}{c} \text{CI}=\frac{\lambda_{\max}-n}{n-1},\\ \text{CR}=\frac{\text{CI}}{\text{RI}}, \end{array}$$

where CI is the consistency index, RI is the random index shown in Table 2, and *n* is the matrix size. As a rule, the consistency of the matrix is considered as acceptable only if CR < 0.10; otherwise the pairwise comparisons must be revised.

#### 2.2.5. Calculate the Fuzzy Weight Vector

Based on the pairwise comparisons in matrix *A*, the weight vectors can be calculated using the following:

(6) $$\begin{array}{c} \alpha_{i}={\left[\prod_{j=1}^{n}a_{ij}^{l}\right]}^{1/n}, \end{array}$$

(7) $$\begin{array}{c} \beta_{i}={\left[\prod_{j=1}^{n}a_{ij}^{m}\right]}^{1/n}, \end{array}$$

(8) $$\begin{array}{c} \gamma_{i}={\left[\prod_{j=1}^{n}a_{ij}^{n}\right]}^{1/n}, \end{array}$$

(9) $$\begin{array}{c} \delta_{i}={\left[\prod_{j=1}^{n}a_{ij}^{s}\right]}^{1/n}, \end{array}$$

(10) $$\begin{array}{c} \alpha =\sum_{i=1}^{n}\alpha_{i}, \end{array}$$

(11) $$\begin{array}{c} \beta =\sum_{i=1}^{n}\beta_{i}, \end{array}$$

(12) $$\begin{array}{c} \gamma =\sum_{i=1}^{n}\gamma_{i}, \end{array}$$

(13) $$\begin{array}{c} \delta =\sum_{i=1}^{n}\delta_{i}, \end{array}$$

(14) $$\begin{array}{c} {\tilde{w}}_{i}=\left(\alpha_{i}\delta^{-1},\beta_{i}\gamma^{-1},\gamma_{i}\beta^{-1},\delta_{i}\alpha^{-1}\right). \end{array}$$

#### 2.2.6. Defuzzification

The crisp value of *w*_*ij*_ can be calculated using defuzzification with the following:

(15) $$\begin{array}{c} w_{ij}=\frac{\alpha_{i}\delta^{-1}+2\beta_{i}\gamma^{-1}+2\gamma_{i}\beta^{-1}+\delta_{i}\alpha^{-1}}{6}. \end{array}$$

### 2.3. Calculate the Scores of the Evaluation Factors

#### 2.3.1. Scores of the Evaluation Factors and Calculating the Fuzzy Evaluation Vectors

Using the data, that is, field survey and sampling data, the fuzzy evaluation vector of a specific factor is calculated as follows. Assume that there are *k* decision-makers *E*_1_, *E*_2_,…, *E*_*k*_, and *n* is attributed to ${\tilde{f}}_{1},{\tilde{f}}_{2},\ldots ,{\tilde{f}}_{n}$. Convert the values into STFN, as shown in Table 3.

#### 2.3.2. Construct the Fuzzy Evaluation Matrix

The evaluation value ${\overset{-}{\tilde{f}}}_{i}$ of the attribute ${\tilde{f}}_{i}$ given by the decision-making group can be obtained as follows:

(16) $$\begin{array}{c} {\tilde{f}}_{i}=\left(f_{i}^{l^{}},f_{i}^{m},f_{i}^{n},f_{i}^{s}\right),\\ {\overset{-}{\tilde{f}}}_{i}=\frac{1}{k}\sum_{i=1}^{k}{\tilde{f}}_{i}. \end{array}$$

### 2.4. Calculate the Evaluation Result

Calculate the risk magnitude (RM) using the following:

(17) $$\begin{array}{c} \text{RM}=w_{ij}\cdot {\overset{-}{\tilde{f}}}_{i}. \end{array}$$

## 3. Case Study

### 3.1. Preliminary Step

The necessary information collected in the preliminary step by the risk assessment group for a specific test case scenario is summarized in Table 4.

### 3.2. Establishment of the FI Stage

Five highly qualified experts in the subject area were selected to form a risk assessment group and they performed the risk assessment using the proposed methodology. Each risk factor was evaluated at the different levels of the FI hierarchy by the experts who awarded scores. Different experts had different weight (Table 5) and they provided precise numerical values, linguistic terms, numerical value ranges, or a fuzzy number based on their knowledge and the information available. These evaluations were converted into STFNs as shown in Table 1 and (6)–(14).

#### 3.2.1. Secondary Indices

Five experts graded the secondary indices (solid waste characteristics, environmental characteristics, tailing dam risk, risk management, and usage) in a pairwise manner according to Table 1 to produce Table 6.

#### 3.2.2. Tertiary Indices

Experts from different fields graded the tertiary indices with which they were familiar in a pairwise manner to produce Table 7.

#### 3.2.3. Quaternary Indices

Experts from different fields graded the quaternary indices with which they were familiar and the results are shown in Table 8.

#### 3.2.4. Matrix Creation and Uniformity Checking

A matrix was created using (1)–(5) and Table 2, and the uniformity of the grades was checked. In this case, CR < 0.10, which demonstrated the uniformity of the grades.

#### 3.2.5. Fuzzy Weight Calculation and Fuzzy Solving

The fuzzy weights were calculated and a fuzzy matrix was produced using (6)–(10). The results are shown in Table 9.

### 3.3. Risk Assessment Stage

Twenty experts graded the risk value according to Table 3. The results are shown in Table 10.

### 3.4. Fuzzy Inference Stage

The assessments were made based on the data in Table 10, which were calculated using (16), and the results are shown in Table 11.

## 4. Discussion

In this study, we developed an ERAS to evaluate the environmental risks of tailing dams produced by the phosphate fertilizer industry in Yunnan. Table 9 shows that five experts gave the most important weighting to the utilization prospects (A5) among the secondary indices. Thus, the utilization prospects had a direct relationship with the environmental risks of tailing dams. Risk management was also important. The leaks and inrushes that occur in tailing dams are caused by inappropriate management. To reduce environmental risks, it is necessary to apply standards and effective management, but the most important task is risk prevention. The ratios of the seepage line and the dry beach length were the major factors used to evaluate the stability of a dam. These were significant dam risk indicators. Acute toxicity reflected the degree of harm due to direct contamination. Radioactivity was also a necessary indicator for PG. Thus, the acute toxicity and radioactivity were given higher weightings than other factors. However, the geography, the population density, and the distance between communities were more important environmental characteristics.

**Figure 3:**
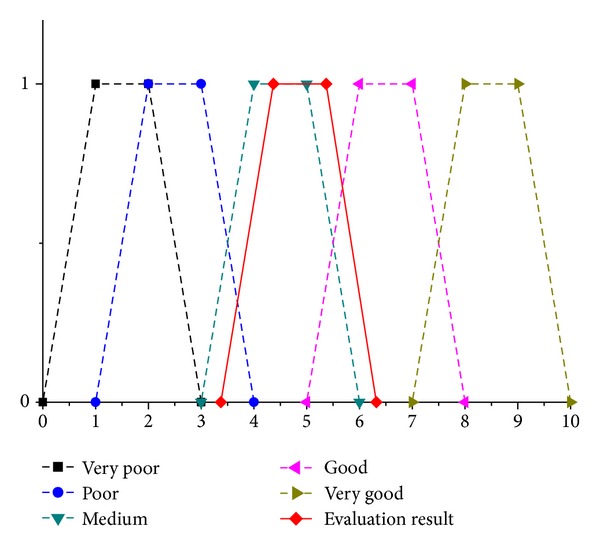
The membership functions of the five grades of the linguistic variables and the evaluation result.

1. As shown in Figure 3, the environmental risk indices for the PG tailing dam were 3.371, 4.370, 5.370, and 6.321, that is, medium, good. Thus, this tailing dam had some risk but could not pollute its surroundings.
2. Table 11 shows that the area around this tailing dam had risk values of 1.25, 2.25, 3.25, and 4.25, which indicated the possible danger of environmental risk due to a pollution source based on the geography. In the next area, there was some risk based on the seepage line and dry beach length ratio, the height, capacity, and material of the dam and the damming mode. Thus, it is necessary to check, rectify, and reform this tailing dam and its surroundings to reduce risk and exclude hidden dangers.

This study established a method for evaluating the environmental risks of typical industrial solid wastes. We used fuzzy logic and AHP to determine the risk of a pollution source. This method is simple to use and can be quantified, so it is a practical management method for decision-makers.

## 5. Conclusions

The establishment of an ERAS for PG tailing dams will help to prevent PG tailing dams from affecting human health. Moreover, this method may help decision-makers who encounter unknown risks during the environmental management of PG tailing dams.

In the present study, we established an ERAS for PG tailing dams using AHP and fuzzy logic. A hierarchical evaluation index system was established, with five factors in the second level, 12 factors in the third level, and 35 factors in the fourth level. STFNs were used to determine the weights of the indices, and fuzzy weight vectors were calculated. The evaluation factors are scored and the fuzzy evaluation vectors were calculated. Finally, a comprehensive solid waste index, environmental index, tailing dam index, risk management index, utilization prospect index, safety grade, and early warning grade were determined. A PG tailing dam was fed into the model to evaluate the work safety performance and determine the safety grade.

This methodology, which combines AHP and fuzzy logic, is a new scientific method for performing environmental risk assessments of PG tailing dams, which generates accurate and comprehensive evaluation results. The safety grade and early warning grade generated by the proposed ERAS method may become powerful tools for officials, managers, and evaluators of PG tailing dams.

## Figures and Tables

**Table 1:** Scale of relative importance used in the pairwise comparison of AHP.

Linguistic variable	Scale of relative importance (crisp number)	Trapezoidal fuzzy number
Equally important	1	(1,1, 1,1)
Weakly important	3	(2,5/2,7/2,4)
Essentially important	5	(4,9/2,11/2,6)
Very strongly important	7	(6,13/2,15/2,8)
Absolutely important	9	(8,17/2,9, 9)
	*x* = 2,4, 6,8 are intermediate scale	(*x* − 1, *x* − 1/2, *x* + 1/2, *x* + 1)

**Table 2:** The random consistency index (RI).

Size (*n*)	1	2	3	4	5	6	7	8	9
RI	0.00	0.00	0.58	0.90	1.12	1.24	1.32	1.41	1.45

**Table 3:** Linguistic variables and trapezoidal fuzzy numbers for the evaluation.

Linguistic variables	Trapezoidal fuzzy numbers
Very poor	(0,1, 2,3)
Poor	(1,2, 3,4)
Medium	(3,4, 5,6)
Good	(5,6, 7,8)
Very good	(7,8, 9,10)

**Table 4:** List for investigation of the tailings of the XX phosphorus chemical industry in Yunnan.

General information	
Total storage capacity	9.8 million m^3^
Starting date	2003.06
Service period	14.86 years
Type of tailings	Valley Type
Dam height	45 m
Storage capacity	8639737 t
Damming mode	Upstream tailings dam
Comprehensive utilization	6.84 × 10^5^ t
Disposed quantity	1.15 × 10^7^ t
Physical-chemical analysis of phosphogypsum	
Element	Content (%)
Water content	24.20
CaO	27.8
Fe_2_O_3_	0.10
Al_2_O_3_	0.53
MgO	0.05
K_2_O	0.13
Na_2_O	0.13
SO_4_^−^	33.9
F	0.49
SiO_2_	11.85
P	0.7
Extraction toxicity	
Index	Unit (mg/L)
pH	5.73
Cu	<0.02
Pb	<0.1
Zn	0.126
Cr	<0.05
Cd	<0.005
Be	<0.005
Ba	4
Ni	<0.04
As	0.0088
Se	0.0004
Ag	<0.01
Hg	0.0015
Cr^6+^	<0.004
Cyanide	<0.001
Fluoride	6.49
Methyl mercury	<10 ng/L
Ethyl mercury	<20 ng/L

**Table 5:** The scale of weight for experts.

Expert	Background	Weight
E_1_	50 years' experience in solid waste management	0.23
E_2_	50 years' experience in environment risk assessment	0.23
E_3_	Mine senior engineer	0.2
E_4_	20 years' experience in tailings dam management	0.18
E_5_	10 years' experience in tailings dam management	0.16

**Table 6:** Scale of relative importance used in the pairwise comparison of AHP for 2 level factors.

		A1		A2		A3		A4		A5	
		Score	Converted STFN	Score	Converted STFN	Score	Converted STFN	Score	Converted STFN	Score	Converted STFN
A1	E_1_	1	(1,1, 1,1)	1	(1,1, 1,1)	1/3	(1/4,2/7,2/5,1/2)	(1,2)	(1,1, 2,2)	1/5	(1/6,2/11,2/9,1/4)
	E_2_	1	(1,1, 1,1)	(1,2)	(1,1, 2,2)	1	(1,1, 1,1)	(1/2,1)	(1/2,1/2,1, 1)	1/3	(1/4,2/7,2/5,1/2)
	E_3_	1	(1,1, 1,1)	1/2	(1/3,2/5,2/3,1)	1/4	(1/5,2/9,2/7,1/3)	1/5	(1/6,2/11,2/9,1/4)	1/7	(1/8,2/15,2/13,1/6)
	E_4_	1	(1,1, 1,1)	1	(1,1, 1,1)	1	(1,1, 1,1)	1	(1,1, 1,1)	1	(1,1, 1,1)
	E_5_	1	(1,1, 1,1)	2	(1,3/2,5/2,3)	1	(1,1, 1,1)	1/3	(1/4,2/7,2/5,1/2)	1/4	(1/5,2/9,2/7,1/3)

Aggregated STFN			(1,1, 1,1)		(0.867,0.960, 1.403,1.550)		(0.668,0.680, 0.719,0.752)		(0.598,0.607, 0.978,1.000)		(0.333,0.350, 0.400,0.439)

A2	E_1_	1	(1,1, 1,1)	1	(1,1, 1,1)	1/2	(1/3,2/5,2/3,1)	(1, 2)	(1,1, 2,2)	1/4	(1/5,2/9,2/7,1/3)
	E_2_	(1/2, 1)	(1/2,1/2,1, 1)	1	(1,1, 1,1)	1	(1,1, 1,1)	1/3	(1/4,2/7,2/5,1/2)	1/5	(1/6,2/11, 2/9,1/4)
	E_3_	2	(1,3/2,5/2,3)	1	(1,1, 1,1)	1/2	(1/3,2/5,2/3,1)	1/3	(1/4,2/7,2/5,1/2)	1/5	(1/6,2/11, 2/9,1/4)
	E_4_	1	(1,1, 1,1)	1	(1,1, 1,1)	1	(1,1, 1,1)	1	(1,1, 1,1)	1	(1,1, 1,1)
	E_5_	1/2	(1/3,2/5,2/3,1)	1	(1,1, 1,1)	1/2	(1/3,2/5,2/3,1)	1/6	(1/7,2/13,2/11,1/5)	1/7	(1/8,2/15, 2/13,1/6)

Aggregated STFN			(0.778,0.889, 1.247,1.400)		(1,1, 1,1)		(0.607,0.646, 0.803,1.000)		(0.540,0.557, 0.841,0.887)		(0.318,0.331, 0.366,0.391)

A3	E_1_	3	(2,5/2,7/2,4)	2	(1,3/2,5/2,3)	1	(1,1, 1,1)	4	(3,7/2,9/2,5)	1/3	(1/4,2/7,2/5,1/2)
	E_2_	1	(1,1, 1,1)	1	(1,1, 1,1)	1	(1,1, 1,1)	1/3	(1/4,2/7,2/5,1/2)	1/3	(1/4,2/7, 2/5,1/2)
	E_3_	4	(3,7/2,9/2,5)	2	(1,3/2,5/2,3)	1	(1,1, 1,1)	1	(1,1, 1,1)	1/2	(1/3,2/5,2/3,1)
	E_4_	1	(1,1, 1,1)	1	(1,1, 1,1)	1	(1,1, 1,1)	1	(1,1, 1,1)	1	(1,1, 1,1)
	E_5_	1	(1,1, 1,1)	2	(1,3/2,5/2,3)	1	(1,1, 1,1)	1/3	(1/4,2/7,2/5,1/2)	1/5	(1/6,2/11,2/9,1/4)

Aggregated STFN			(1.630,1.845, 2.275,2.490)		(1.000,1.295, 1.885,2.180)		(1,1, 1,1)		(1.168,1.296, 1.571,1.725)		(0.388,0.421,0.533, 0.650)

A4	E_1_	(1/2,1)	(1/2,1/2,1, 1)	(1/2,1)	(1/2,1/2,1, 1)	1/4	(1/5,2/9,2/7,1/3)	1	(1,1, 1,1)	1/6	(1/7,2/13, 2/11,1/5)
	E_2_	(1,2)	(1,1, 2,2)	3	(2,5/2,7/2,4)	3	(2,5/2,7/2,4)	1	(1,1, 1,1)	1/2	(1/3,2/5,2/3,1)
	E_3_	5	(4,9/2,11/2,6)	3	(2,5/2,7/2,4)	1	(1,1, 1,1)	1	(1,1, 1,1)	1/2	(1/3,2/5,2/3,1)
	E_4_	1	(1,1, 1,1)	1	(1,1, 1,1)	1	(1,1, 1,1)	1	(1,1, 1,1)	1	(1,1, 1,1)
	E_5_	3	(2,5/2,7/2,4)	6	(5,11/2,13/2,7)	3	(2,5/2,7/2,4)	1	(1,1, 1,1)	(1/2,1)	(1/2,1/2,1, 1)

Aggregated STFN			(1.645,1.825, 2.530,2.710)		(1.955,2.250, 2.955,3.250)		(1.206,1.406, 1.811,2.017)		(1,1, 1,1)		(0.436,0.467, 0.668,0.816)

A5	E_1_	5	(4,9/2,11/2,6)	4	(3,7/2,9/2,5)	3	(2,5/2,7/2,4)	6	(5,11/2,13/2,7)	1	(1,1, 1,1)
	E_2_	3	(2,5/2,7/2,4)	5	(4,9/2,11/2,6)	3	(2,5/2,7/2,4)	2	(1,3/2,5/2,3)	1	(1,1, 1,1)
	E_3_	7	(6,13/2,15/2,8)	5	(4,9/2,11/2,6)	2	(1,3/2,5/2,3)	2	(1,3/2,5/2,3)	1	(1,1, 1,1)
	E_4_	1	(1,1, 1,1)	1	(1,1, 1,1)	1	(1,1, 1,1)	1	(1,1, 1,1)	1	(1,1, 1,1)
	E_5_	4	(3,7/2,9/2,5)	7	(6,13/2,15/2,8)	5	(4,9/2,11/2,6)	(1, 2)	(1,1, 2,2)	1	(1,1, 1,1)

Aggregated STFN			(3.240,3.650, 4.470,4.880)		(3.550,3.960, 4.780,5.190)		(1.940,2.350, 3.170,3.580)		(1.920,2.250, 3.070,3.400)		(1,1, 1,1)

**Table:** (a)

	B1	B2
B1	(1,1, 1,1)	(1/4,2/7,2/5,1/2)
B2	(2,5/2,7/2,4)	(1,1, 1,1)

**Table:** (b)

	B3	B4
B3	(1,1, 1,1)	(1,1, 1,1)
B4	(1,1, 1,1)	(1,1, 1,1)

**Table:** (c)

	B5	B6	B7
B5	(1,1, 1,1)	(2,5/2,7/2,4)	(2,5/2,7/2,4)
B6	(1/4,2/7,2/5,1/2)	(1,1, 1,1)	(1,1, 1,1)
B7	(1/4,2/7,2/5,1/2)	(1,1, 1,1)	(1,1, 1,1)

**Table:** (d)

	B8	B9
B8	(1,1, 1,1)	(1,3/2,5/2,3)
B9	(1/3,2/5,2/3,1)	(1,1, 1,1)

**Table:** (e)

	B10	B11	B12
B10	(1,1, 1,1)	(1,1, 1,1)	(1,1, 1,1)
B11	(1,1, 1,1)	(1,1, 1,1)	(1,1, 1,1)
B12	(1,1, 1,1)	(1,1, 1,1)	(1,1, 1,1)

**Table:** (a)

	C1	C2	C3
C1	(1,1, 1,1)	(1/4,2/7,2/5,1/2)	(1/7,2/13,2/11,1/5)
C2	(2,5/2,7/2,4)	(1,1, 1,1)	(1/3,1/3,1/2,1/2)
C3	(5,11/2,13/2,7)	(2,2, 3,3)	(1,1, 1,1)

**Table:** (b)

	C4	C5	C6	C7
C4	(1,1, 1,1)	(1,3/2,5/2,3)	(1/6,2/11,2/9,1/4)	(1/5,2/9,2/7,1/3)
C5	(1/3,2/5,2/3,1)	(1,1, 1,1)	(1/7,2/13,2/11,1/5)	(1/6,2/11,2/9,1/4)
C6	(4,9/2,11/2,6)	(5,11/2,13/2,7)	(1,1, 1,1)	(1,1, 2,2)
C7	(3,7/2,9/2,5)	(4,9/2,11/2,6)	(1/2,1/2,1, 1)	(1,1, 1,1)

**Table:** (c)

	C8	C9	C10	C11	C12
C8	(1,1, 1,1)	(1/6,2/11,2/9,1/4)	(1/8,2/15,2/13,1/6)	(1/6,2/11,2/9,1/4)	(1/3,2/5,2/3,1)
C9	(4,9/2,11/2,6)	(1,1, 1,1)	(1/3,2/5,2/3,1)	(1,1, 1,1)	(2,5/2,7/2,4)
C10	(6,13/2,15/2,8)	(1,3/2,5/2,3)	(1,1, 1,1)	(1,3/2,5/2,3)	(5,11/2,13/2,7)
C11	(4,9/2,11/2,6)	(1,1, 1,1)	(1/3,2/5,2/3,1)	(1,1, 1,1)	(2,5/2,7/2,4)
C12	(1,3/2,5/2,3)	(1/4,2/7,2/5,1/2)	(1/7,2/13,2/11,1/5)	(1/4,2/7,2/5,1/2)	(1,1, 1,1)

**Table:** (d)

	C13	C14	C15	C16	C17	C18
C13	(1,1, 1,1)	(5,11/2,13/2,7)	(4,9/2,11/2,6)	(4,9/2,11/2,6)	(4,9/2,11/2,6)	(1/4,2/7,2/5,1/2)
C14	(1/7,2/13,2/11,1/5)	(1,1, 1,1)	(1/4,2/7,2/5,1/2)	(1/4,2/7,2/5,1/2)	(1/4,2/7,2/5,1/2)	(1/9,2/17,2/15,1/7)
C15	(1/6,2/11,2/9,1/4)	(2,5/2,7/2,4)	(1,1, 1,1)	(1,1, 1,1)	(1,1, 1,1)	(1/7,2/13,2/11,1/5)
C16	(1/6,2/11,2/9,1/4)	(2,5/2,7/2,4)	(1,1, 1,1)	(1,1, 1,1)	(1,1, 1,1)	(1/7,2/13,2/11,1/5)
C17	(1/6,2/11,2/9,1/4)	(2,5/2,7/2,4)	(1,1, 1,1)	(1,1, 1,1)	(1,1, 1,1)	(1/7,2/13,2/11,1/5)
C18	(2,5/2,7/2,4)	(7,15/2,17/2,9)	(5,11/2,13/2,7)	(5,11/2,13/2,7)	(5,11/2,13/2,7)	(1,1, 1,1)

**Table:** (e)

	C19	C20	C21	C22	C23	C24
C19	(1,1, 1,1)	(4,9/2,11/2,6)	(2,5/2,7/2,4)	(4,9/2,11/2,6)	(4,9/2,11/2,6)	(2,5/2,7/2,4)
C20	(1/6,2/11,2/9,1/4)	(1,1, 1,1)	(1/4,2/7,2/5,1/2)	(1,1, 1,1)	(1,1, 1,1)	(1/4,2/7,2/5,1/2)
C21	(1/4,2/7,2/5,1/2)	(2,5/2,7/2,4)	(1,1, 1,1)	(2,5/2,7/2,4)	(2,5/2,7/2,4)	(1,1, 1,1)
C22	(1/6,2/11,2/9,1/4)	(1,1, 1,1)	(1/4,2/7,2/5,1/2)	(1,1, 1,1)	(1,1, 1,1)	(1/4,2/7,2/5,1/2)
C23	(1/6,2/11,2/9,1/4)	(1,1, 1,1)	(1/4,2/7,2/5,1/2)	(1,1, 1,1)	(1,1, 1,1)	(1/4,2/7,2/5,1/2)
C24	(1/4,2/7,2/5,1/2)	(2,5/2,7/2,4)	(1,1, 1,1)	(2,5/2,7/2,4)	(2,5/2,7/2,4)	(1,1, 1,1)

**Table:** (f)

	C25	C26	C27
C25	(1,1, 1,1)	(1,1, 1,1)	(1,3/2,5/2,3)
C26	(1,1, 1,1)	(1,1, 1,1)	(1,3/2,5/2,3)
C27	(1/3,2/5,2/3,1)	(1/3,2/5,2/3,1)	(1,1, 1,1)

**Table:** (g)

	C28	C29
C28	(1,1, 1,1)	(1,1, 1,1)
C29	(1,1, 1,1)	(1,1, 1,1)

**Table:** (h)

	C30	C31	C32	C33
C30	(1,1, 1,1)	(1/4,2/7,2/5,1/2)	(1/5,2/9,2/7,1/3)	(1/6,2/11,2/9,1/4)
C31	(2,5/2,7/2,4)	(1,1, 1,1)	(1/3,2/5,2/3,1)	(1/4,2/7,2/5,1/2)
C32	(3,7/2,9/2,5)	(1,3/2,5/2,3)	(1,1, 1,1)	(1/3,2/5,2/3,1)
C33	(4,9/2,11/2,6)	(2,5/2,7/2,4)	(1,3/2,5/2,3)	(1,1, 1,1)

**Table:** (i)

	C34	C35
C34	(1,1, 1,1)	(1/4,2/7,2/5,1/2)
C35	(2,5/2,7/2,4)	(1,1, 1,1)

**Table 9:** The weights of the factors and subfactors.

	Fuzzy weight vector	Defuzzified weights
Characteristics of solid waste (A1)	(0.081,0.091,0.139,0.161)	0.112
Characteristics of environment (A2)	(0.075,0.087,0.132,0.159)	0.108
Tailing dam risk (A3)	(0.117,0.143,0.216,0.264)	0.176
Risk management (A4)	(0.138,0.165,0.260,0.315)	0.209
Utilization prospect (A5)	(0.264,0.323,0.487,0.580)	0.395
Physical characteristic (B1)	(0.185,0.214,0.299,0.369)	0.256
Chemical characteristic (B2)	(0.522,0.632,0.884,1.045)	0.744
Natural environment (B3)	(0.5, 0.5, 0.5, 0.5)	0.5
Social environment (B4)	(0.5, 0.5, 0.5, 0.5)	0.5
Characteristics of dam body (B5)	(0.386,0.487,0.730,0.885)	0.598
Drainage installation (B6)	(0.153,0.174,0.233,0.279)	0.201
Flood drainage facility (B7)	(0.153,0.174,0.233,0.279)	0.201
Risk prevention (B8)	(0.366,0.511,0.851,1.098)	0.650
Emergency response (B9)	(0.211,0.264,0.440,0.634)	0.350
Support of government (B10)	(1/3, 1/3, 1/3, 1/3)	1/3
Using proportion (B11)	(1/3, 1/3, 1/3, 1/3)	1/3
Technology maturity (B12)	(1/3, 1/3, 1/3, 1/3)	1/3
Volatility (C1)	(0.073,0.082,0.119,0.138)	0.100
Solubility (C2)	(0.195,0.218,0.343,0.375)	0.274
Radioactivity (C3)	(0.481,0.516,0.765,0.822)	0.626
Corrosivity (C4)	(0.065,0.080,0.134,0.161)	0.104
Acidity and alkaline (C5)	(0.046,0.053,0.086,0.107)	0.068
Acute toxicity (C6)	(0.323,0.361,0.615,0.687)	0.470
Extraction toxicity (C7)	(0.239,0.271,0.472,0.531)	0.358
Air (C8)	(0.039,0.046,0.073,0.094)	0.053
Soil (C9)	(0.127,0.152,0.351,0.443)	0.230
Geology (C10)	(0.172,0.231,0.661,0.815)	0.403
Hydrology (C11)	(0.127,0.152,0.351,0.443)	0.230
Ecology (C12)	(0.047,0.061,0.125,0.161)	0.084
Population density (C13)	(0.223,0.255,0.332,0.379)	0.291
Industry (C14)	(0.031,0.035,0.049,0.060)	0.043
Agriculture (C15)	(0.071,0.079,0.099,0.112)	0.088
Tourism (C16)	(0.071,0.079,0.099,0.112)	0.088
Animal husbandry (C17)	(0.071,0.079,0.099,0.112)	0.088
Distance of community (C18)	(0.296,0.346,0.464,0.537)	0.402
Seepage line and dry beach length (C19)	(0.270,0.339,0.514,0.631)	0.417
Height and capacity (C20)	(0.050,0.058,0.083,0.102)	0.069
Damming mode (C21)	(0.120,0.151,0.232,0.289)	0.188
Type of tailings (C22)	(0.050,0.058,0.083,0.102)	0.069
Service period (C23)	(0.050,0.058,0.083,0.102)	0.069
Material of dam (C24)	(0.120,0.151,0.232,0.289)	0.188
Returning reservoir (C25)	(0.257,0.329,0.479,0.581)	0.390
Seepage shaft (C26)	(0.257,0.329,0.479,0.581)	0.390
Drainage (C27)	(0.124,0.156,0.269,0.403)	0.220
Flood discharge trench (C28)	(0.5, 0.5, 0.5, 0.5)	0.5
Flood draining facility (C29)	(0.5, 0.5, 0.5, 0.5)	0.5
Revetment construction maintenance (C30)	(0.046,0.058,0.093,0.125)	0.073
Dam safety monitoring (C31)	(0.098,0.129,0.229,0.328)	0.175
Daily management (C32)	(0.153,0.212,0.386,0.543)	0.291
ISO authentication (C33)	(0.258,0.357,0.614,0.804)	0.461
Emergency capacity of rescuing facilities (C34)	(0.185,0.214,0.299,0.369)	0.256
Capacity of emergency protection and eliminating leak (C35)	(0.522,0.632,0.884,1.045)	0.744

**Table 10:** The summarization of the initial date of the evaluation for the 4-level factors.

	VP	P	M	G	VG
C1	0	3	11	5	1
C2	1	3	7	8	1
C3	0	2	5	10	3
C4	3	10	5	2	0
C5	2	11	6	1	0
C6	1	4	8	5	2
C7	0	1	7	11	1
C8	0	3	9	8	0
C9	3	7	7	3	0
C10	5	10	5	0	0
C11	1	4	10	3	2
C12	1	1	8	7	3
C13	1	1	8	8	2
C14	1	2	10	2	1
C15	0	3	6	9	2
C16	0	0	8	8	4
C17	0	2	9	7	2
C18	1	2	5	9	3
C19	1	5	10	4	0
C20	1	4	11	4	0
C21	3	8	6	3	0
C22	1	3	12	3	1
C23	1	1	8	7	3
C24	2	6	10	1	1
C25	0	0	8	10	2
C26	0	1	8	9	2
C27	1	2	9	6	2
C28	1	2	10	6	1
C29	2	3	8	5	2
C30	1	1	6	10	2
C31	0	1	7	11	1
C32	0	1	6	12	1
C33	0	0	8	11	1
C34	1	1	6	10	2
C35	1	2	7	9	1
B10	1	2	10	7	0
B11	2	4	10	3	1
B12	1	3	11	4	1

**Table 11:** The evaluation results.

	The fuzzy evaluating vectors	Rating
Volatility (C1)	(3.4,4.4,5.4,6.4)	(M, G)
Solubility (C2)	(3.55,4.55,5.55, 6.55)	(M, G)
Radioactivity (C3)	(4.4,5.4,6.4,7.4)	(M, G)
Corrosivity (C4)	(1.75,2.75,3.75,4.75)	(P, M)
Acidity and alkaline (C5)	(1.7,2.7,3.7,4.7)	(P, M)
Acute toxicity (C6)	(3.35,4.35,5.35,6.35)	(M, G)
Extraction toxicity (C7)	(4.2,5.2,6.2,7.2)	(M, G)
Air (C8)	(3.5,4.5,5.5,6.5)	(M, G)
Soil (C9)	(2.15,3.15,4.15,3.95)	(P, M)
Geology (C10)	(1.25,2.25,3.25,4.25)	(P, M)
Hydrology (C11)	(3.15,4.15,5.15,6.15)	(M, G)
Ecology (C12)	(4.05,5.05,6.05,7.05)	(M, G)
Population density (C13)	(3.95,4.95,5.95,6.95)	(M, G)
Industry (C14)	(2.45,3.25,4.05,4.85)	(P, M)
Agriculture (C15)	(4,5, 6,7)	(M, G)
Tourism (C16)	(4.6,5.6,6.6,7.6)	(M, G)
Animal husbandry (C17)	(3.9,4.9,5.9,6.9)	(M, G)
Distance of community (C18)	(4.15,5.15,6.15,7.15)	(M, G)
Seepage line and dry beach length (C19)	(2.75,3.75,4.75,5.75)	(P, M)
Height and capacity (C20)	(2.85,3.85,4.85,5.85)	(P, M)
Damming mode (C21)	(2.05,3.05,4.05,5.05)	(P, M)
Type of tailings (C22)	(3.05,4.05,5.05,6.05)	(M, G)
Service period (C23)	(4.05,5.05,6.05,7.05)	(M, G)
Material of dam (C24)	(2.4,3.4,4.4,5.4)	(P, M)
Returning reservoir (C25)	(4.4,5.4,6.4,7.4)	(M, G)
Seepage shaft (C26)	(4.2,5.2,6.2,7.2)	(M, G)
Drainage (C27)	(3.65,4.65,5.65,6.65)	(M, G)
Flood discharge trench (C28)	(3.45,4.45,5.45,6.45)	(M, G)
Flood draining facility (C29)	(3.3,4.3,5.3,6.3)	(M, G)
Revetment construction maintenance (C30)	(4.15,5.15,6.15,7.15)	(M, G)
Dam safety monitoring (C31)	(4.2,5.2,6.2,7.2)	(M, G)
Daily management (C32)	(4.3, 5.3,6.3,7.3)	(M, G)
ISO authentication (C33)	(4.3,5.3,6.3,7.3)	(M, G)
Emergency capacity of rescuing facilities (C34)	(4.15,5.15,6.15,5.35)	(M, G)
Capacity of emergency protection and eliminating leak (C35)	(3.75,4.75,5.75,6.75)	(M, G)

Support of government (B10)	(3.35,4.35,5.35,6.35)	(M, G)
Using proportion (B11)	(2.8,3.8,4.8,5.8)	(P, M)
Technology maturity (B12)	(3.15,4.15,5.15,6.15)	(M, G)
Physical characteristic (B1)	(4.067,5.067,6.067,7.067)	(M, G)
Chemical characteristic (B2)	(3.376,4.376,5.376,6.376)	(M, G)
Natural environment (B3)	(2.248,3.248,4.248,4.972)	(P, M)
Social environment (B4)	(4.023,5.015,6.006,6.997)	(M, G)
Characteristics of dam body (B5)	(2.670,3.670,4.670,5.670)	(P, M)
Drainage installation (B6)	(4.157,5.157,6.157,7.157)	(M, G)
Flood drainage facility (B7)	(3.375,4.375,5.375,6.375)	(M, G)
Risk prevention (B8)	(4.272,5.272,6.272,7.272)	(M, G)
Emergency response (B9)	(3.852,4.852,5.852,6.392)	(M, G)

Characteristics of solid waste (A1)	(3.553,4.553,5.553,6.553)	(M, G)
Characteristics of environment (A2)	(3.136,4.131,5.127,5.985)	(M, G)
Tailing dam risk (A3)	(3.111,4.111,5.111,6.111)	(M, G)
Risk management (A4)	(4.125,5.125,6.125,6.964)	(M, G)
Utilization prospect (A5)	(3.100,4.100,5.100,6.100)	(M, G)

Final objective	(3.371,4.370,5.370,6.321)	(M, G)
